# Value of KPNA4 as a diagnostic and prognostic biomarker for hepatocellular carcinoma

**DOI:** 10.18632/aging.202447

**Published:** 2021-02-01

**Authors:** Mingxing Xu, Hao Liang, Kun Li, Shu Zhu, Zhicheng Yao, Ruiyun Xu, Nan Lin

**Affiliations:** 1 Department of Hepatobiliary Surgery, The Third Affiliated Hospital of Sun Yat-sen University, Guangzhou 510630, Guangdong, China; 2 Department of General Surgery, The Third Affiliated Hospital of Sun Yat-sen University, Guangzhou 510630, Guangdong, China; 3 Department of Liver Transplantation, The Third Affiliated Hospital of Sun Yat-sen University, Guangzhou 510630, Guangdong, China; 4 Department of Infectious Diseases, The Third Affiliated Hospital of Sun Yat-sen University, Guangzhou 510630, Guangdong, China

**Keywords:** KPNA4, hepatocellular carcinoma, immune infiltration, overall survival, biomarker

## Abstract

It is important to identify novel biomarkers to improve hepatocellular carcinoma (HCC) diagnosis and treatment. Herein, we reported the role of karyopherin α4 (KPNA4) in HCC patients through public data mining and examined the results using clinical samples in our center. Our results revealed that KPNA4 expression level was positively correlated with the infiltration of CD8^+^ T cells, B cells, dendritic cells, CD4^+^ T cells, neutrophils and macrophages. In addition, KPNA4 expression was significantly associated with T cell exhaustion. KPNA4 mRNA and protein expression levels were significantly higher in cancerous tissue than in normal tissue. Besides, the increased expression of KPNA4 indicated poor overall survival. Univariate and multivariate Cox regression analyses showed KPNA4 could be viewed as an independent risk factor for HCC patients. Moreover, our experimental results were consistent with those obtained from bioinformatic results. These findings revealed KPNA4 may serve as a novel prognostic biomarker and a potential therapeutic target for HCC.

## INTRODUCTION

Hepatocellular carcinoma (HCC) is one of the most common fatal cancers worldwide. Although the screening method of HCC improves, the early detection rate is still unsatisfactory [1]. Identification of novel biomarkers of HCC may assist in the early diagnosis and treatment, and subsequently improve outcomes.

Karyopherin α (KPNA) are a type of carriers that mediate the shuttling of many transcription factors, and has been proved to be involved in numerous tumors [2–5]. KPNA4 is an importin which had been linked to a number of malignancies. For example, it has been reported that KPNA4 is closely associated with prostate cancer metastasis [6]. Wang et al. reported that KPNA4 attenuates the epithelial-mesenchymal transition (EMT) inhibitory effect of miR-181b in glioblastoma [7]. In cutaneous squamous cell carcinoma, KPNA4 has been shown to promote cancer cell proliferation and cisplatin resistance [8]. The significances of KPNA4 in HCC, however, have not been explored.

Herein, the authors explored the roles of KPNA4 in HCC, with the aim of identifying a novel biomarker for HCC.

## RESULTS

### Correlation of KPNA4 expression with immune cells in HCC

**Figure 1:**
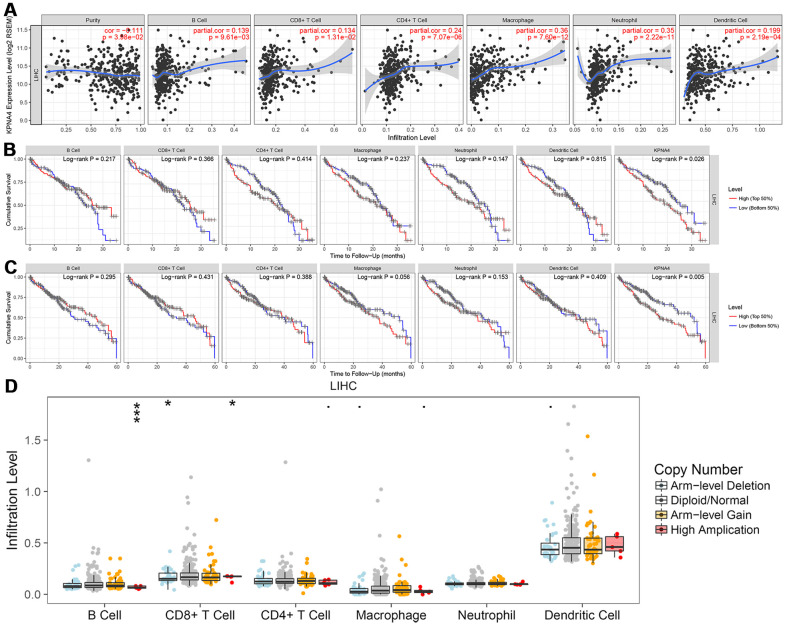
**Correlation between KPNA4 expression and immune infiltration in HCC.** (**A**) The correlation between KPNA4 expression and immune infiltrations in HCC. (**B**, **C**) 3-year and 5-year survival analysis of immune infiltrations and KPNA4 expression in HCC patients. (**D**) Somatic copy number alterations analysis of infiltrated immune cells in HCC.

The TIMER database was used to examine the correlation between KPNA4 and immune infiltration, and it could be found that the levels of infiltration of CD8^+^ T cells, B cells, dendritic cells, CD4^+^ T cells, and neutrophils and macrophages were positively associated with KPNA4 levels (Figure 1A, all P < 0.05).

Then, we used multivariable Cox regression to determine the clinical relevance of immune cell levels with respect to clinical parameters and KPNA4 expression. The results showed that tumor stage, macrophage, and dendritic cell levels, and KPNA4 expression were all significant risk factors for a poor prognosis and KPNA4 expression was an independent factor that influenced 3-year and 5-year survival rates (Figure 1B, 1C and Table 1).

**Table 1:** Cox regression analysis of immune infiltration, KPNA4 expression, and clinical parameters of 315 HCC patients in the TIMER database.

**Variable**	**Coefficient**	**HR**	**95% CI_low**	**95% CI_up**	**P-value**
Age	0.015	1.016	0.999	1.032	0.066
Male	0.012	1.012	0.650	1.576	0.957
Stage2	0.305	1.357	0.814	2.263	0.242
Stage3	0.747	2.110	1.320	3.373	0.002
Stage4	1.383	3.988	1.166	13.647	0.028
Purity	0.977	2.657	0.940	7.513	0.065
B cell	-4.375	0.013	0.000	15.644	0.229
CD8^+^ T cell	-4.465	0.012	0.000	1.677	0.079
CD4^+^ T cell	-5.600	0.004	0.000	6.286	0.140
Macrophage	6.719	828.020	2.900	236438.278	0.020
Neutrophil	-3.886	0.021	0.000	2129.074	0.510
Dendritic	3.910	49.909	1.232	2021.762	0.038
KPNA4	0.818	2.265	1.246	4.116	0.007

CI: confidence interval; HR: hazard ratio.

**Figure 2:**
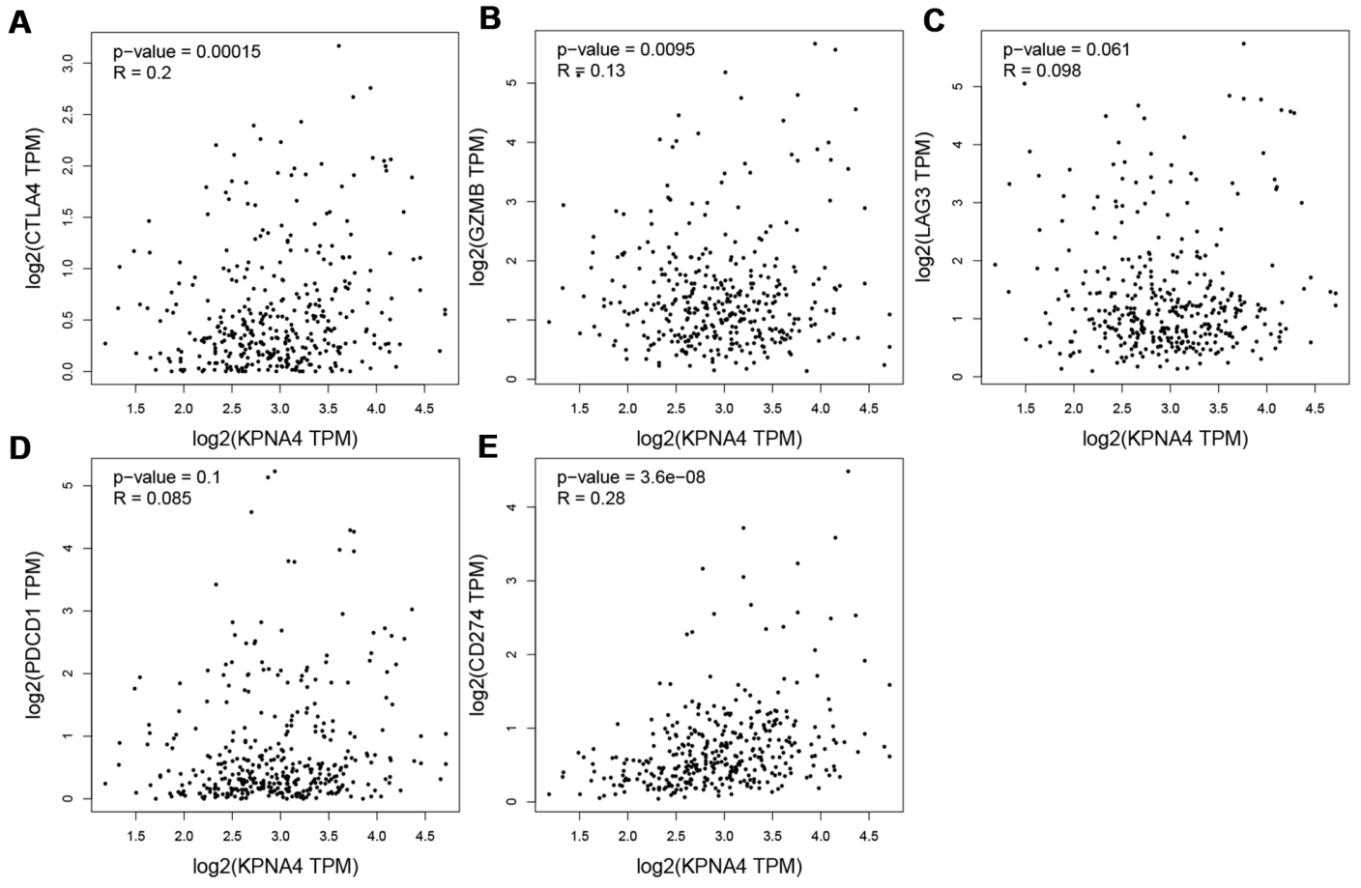
**Correlation between KPNA4 expression and T cell exhaustion in HCC.** (**A**–**E**) Correlation analysis between KPNA4 and related gene markers (CTLA4, GZMB, LAG3 and PDCD1) of T cell exhaustion and PDL1 (CD274) expression.

Somatic copy number alteration (SNCA) analysis indicated that high amplification category of infiltrated B cells in HCC was significantly different with that in normal samples, arm-level deletion and high amplification of infiltrated CD8^+^ T cells were the two categories which significantly altered in HCC (Figure 1D). In addition, we used the GEPIA database to examine correlations of KPNA4 and T cell exhaustion markers PD-1 (PDCD1), GZMB, LAG3, and CTLA4 in cancerous tissues, and to examine the correlation between KPNA4 and PDL1 (CD274). The results indicated a positive correlation between KPNA4 expression and CTLA4, GZMB, and PDL1 expressions in cancerous tissues (Figure 2A, 2B, 2E, all P < 0.05), but no significant correlations with other markers (Figure 2C, 2D).

### KPNA4 expression in the HPA and co-expression networks in HCCDB

**Figure 3:**
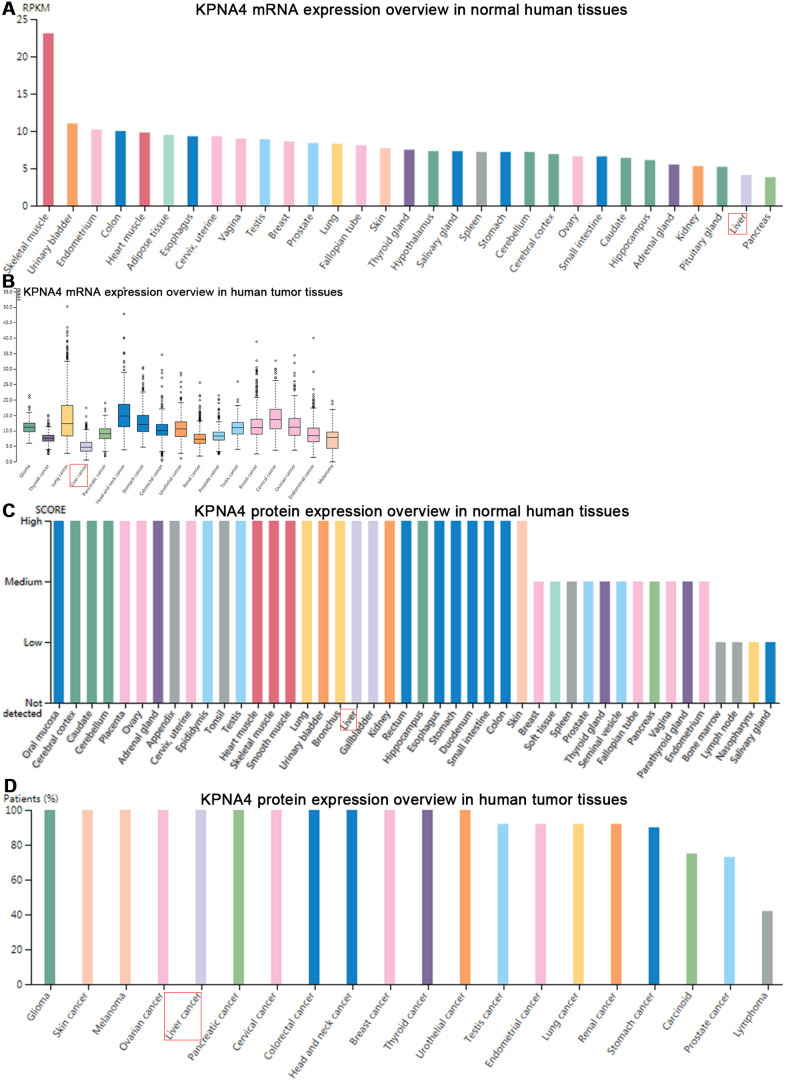
**Gene and protein expression profiles of KPNA4 in human normal and tumor tissues in HPA database.** (**A**) mRNA expression data from the GTEx project. (**B**) Gene expression in common human tumor tissues. (**C**) Protein expression of normal tissues in different organs. (**D**) Protein expression overview in common tumors.

Figure 3A showed the KPNA4 expression features in the HPA, and a relatively low mRNA level could be found in normal liver tissues compared with other organ tissues. Next, we used this database to determine the difference of KPNA4 mRNA expression in various cancers, and found that HCC is the lowest expression type among them (Figure 3B). However, as shown in Figure 3C, 3D, KPNA4 protein expression in the liver presents a higher level compared with that in other organs. In addition, in A-431, PC-3, and U-2 OS cell lines, it could be seen that KPNA4 localization was enriched in the nucleus (Supplementary Figure 1).

**Figure 4:**
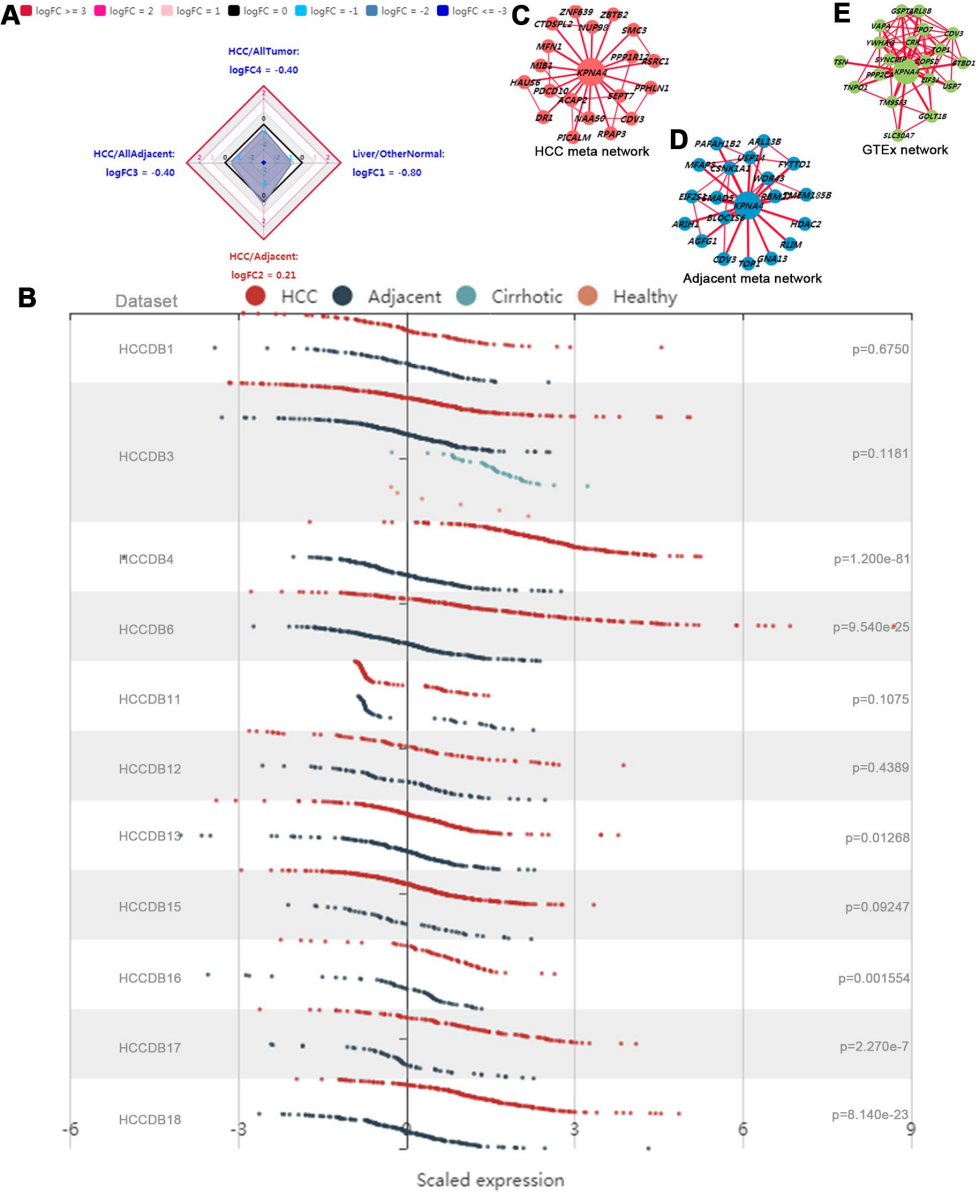
**Gene expression profiles in HCCDB database.** (**A**) Radar map of KPNA4 overall expression among different types of tissues. (**B**) The differential expression of KPNA4 in different liver cancer datasets. The coexpression networks of KPNA4 in HCC tissues (**C**), adjacent liver tissues (**D**) and normal tissues from the GTEx project (**E**) showed that different liver tissues expressed different coexpression networks.

The KPNA4 expression overview among various tissues was illustrated with a radar chart. As shown in Figure 4A, KPNA4 expression in normal liver tissue was lower than that in other normal tissues (liver/other normal: logFC = -0.80); similarly, in HCC tissue, the level was lower than other cancerous samples (HCC/all tumor: logFC = -0.40). However, when compared with adjacent normal liver tissue, it could be found that KPNA4 level was much higher in HCC (logFC = 0.21).

Next, KPNA4 differential expressions were explored in 11 datasets from the HCCDB. It could be found that KPNA4 level in HCC was much higher than that in adjacent liver tissue among 6 of 11 datasets (HCCDB4, HCCDB6, HCCDB13, HCCDB16, HCCDB17 and HCCDB18) (Figure 4B). Results of the KPNA4 co-expression networks indicated there have been vastly different co-expression modes among different tissues (HCC tissue, adjacent tissue, and normal liver tissue) (Figure 4C–4E).

### Association of KPNA4 expression with clinical parameters and influence on patient survival

**Figure 5:**
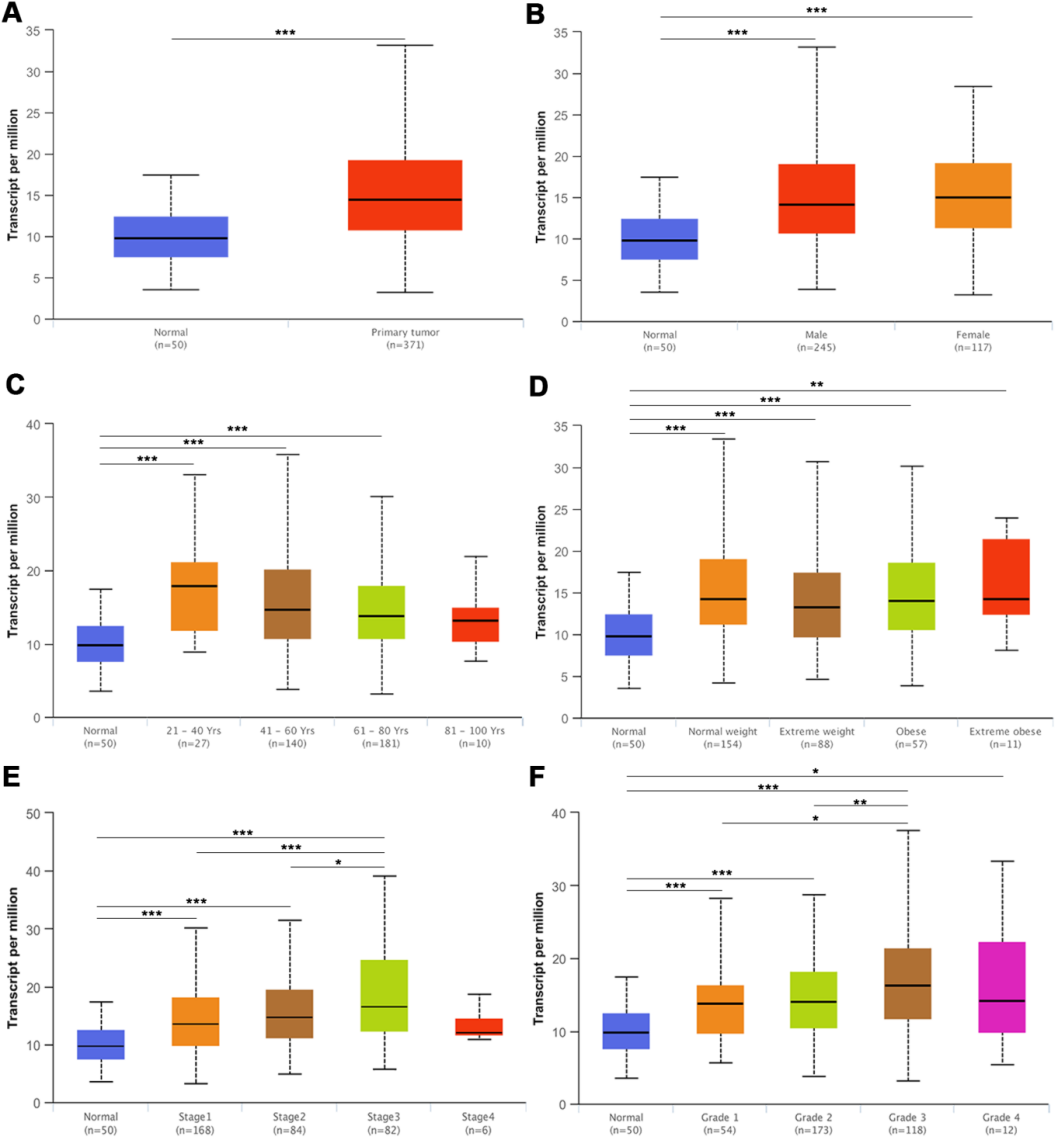
**The mRNA expression of KPNA4 in the UALCAN database.** (**A**) The mRNA expression level of KPNA4 was significantly higher in cancer tissues than in normal tissues. (**B**) The expression level of KPNA4 in HCC patients was higher than that in healthy people and was not associated with gender. (**C**) Patients (age > 21 years old) commonly had higher gene expression than young healthy people. (**D**) The expression level of KPNA4 was not correlated with patient weight. The expression level of KPNA4 was positively correlated with tumor stage (**E**) and tumor grade (**F**) in HCC. * represents P < 0.05, ** represents P < 0.01, *** represents P < 0.001.

As indicated above, the expression of KPNA4, at both mRNA and protein levels, was much higher in HCC samples than normal samples. Next, we employed the UALCAN database to determine the correlations between KPNA4 and clinical indices. As shown in Figure 5A, KPNA4 mRNA expression was higher in HCC than that in normal samples, and meanwhile there have been a higher level in HCC patients than healthy individuals (Figure 5B). Besides, patients > 21 years old generally had higher KPNA4 level compared to those < 21 years old healthy individuals (Figure 5C). However, KPNA4 expression was not correlated with patient weight (Figure 5D). Lastly, tumor stage and grade were analyzed in HCC patients, and it can be found that increased KPNA4 mRNA expression was associated with higher tumor stage and grade (Figure 5E, 5F).

**Figure 6:**
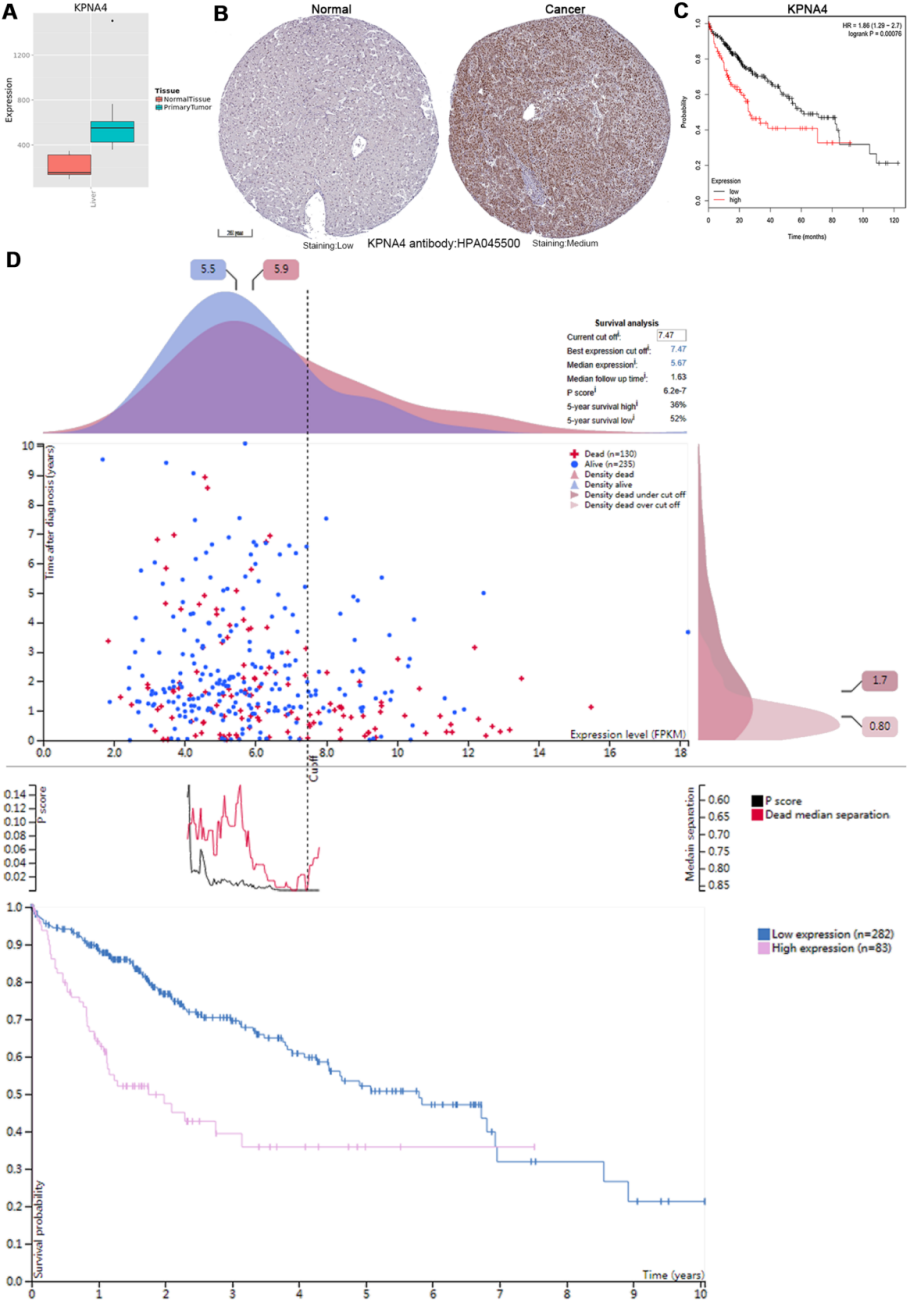
**KPNA4’s expression and prognostic value in HCC patients.** (**A**) The mRNA expression level of KPNA4 was significantly higher in cancerous tissues than in normal tissues in the Metabolic gEne RApid Visualizer. (**B**) Representative immunohistochemistry images from the HPA with the KPNA4 antibody: HPA045500, cancerous tissue had higher staining than normal liver tissue. (**C**) High KPNA4 mRNA expression was significantly associated with poor OS in HCC patients from the Kaplan-Meier plotter. (**D**) Prognostic value of the KPNA4 level in HCC patients from the HPA.

As shown in Figure 6A, 6B, KPNA4 expressions in normal samples were lower compared with HCC samples. Then, Kaplan-Meier plotter was used to examine the correlation between KPNA4 and patient survival, and it could be concluded that KPNA4 expression was negatively correlated with patient survival (Figure 6C, hazard ratio (HR) = 1.86, 95% confidence interval (CI): 1.29-2.7, P = 0.00076). Furthermore, we performed a survival analysis in the HPA to confirm our findings, and the results were consistent with those presented above (Figure 6D).

### KPNA4 expression is an independent risk factor for the prognosis of HCC patients

Firebrowse website (http://firebrowse.org/api-docs/) was used to download data of KPNA4 expression and clinical parameters, and after obtaining the data, Cox survival analysis was performed. By examining data integrity and professional screening, we included 210 patients with 13 clinical parameters for this analysis (Table 2). Table 3 showed the results of univariate and multivariate analyses, and it could be found that in univariate analysis, body mass index (BMI) (HR = 1.037, 95% CI: 1.007-1.068, P = 0.015), total bilirubin (TB) level (HR = 0.040, 95% CI: 0.004-0.438, P = 0.008), and KPNA4 mRNA level (HR = 1.001, 95% CI: 1.000-1.001, P = 0.027) were identified as significant factors influencing overall survival (OS); age (HR = 1.034, 95% CI: 1.004-1.065, P = 0.026), BMI (HR = 1.037, 95% CI: 1.004-1.070, P = 0.026), TB level (HR = 0.038, 95% CI: 0.002-0.767, P = 0.033), tumor histopathological grade (HR = 1.819, 95% CI: 1.132-2.921, P = 0.013) and KPNA4 mRNA level (HR = 1.001, 95% CI: 1.000-1.002, P = 0.012) were significantly associated with OS in multivariate analysis.

**Table 2:** Clinicopathological variables of 210 HCC patients with complete data.

**Variables**	**HCC patients (N=210)**
Gender (Male/female)	141/69
Age (years, Mean±SD)	58.96±12.48
BMI	26.48±9.55
Albumin (g/L, Median)	4.00(0.20-5200)
AFP (ng/ml, Median)	17.00(1-2035000)
Platelets (10e9/L, Median)	200.5(69-499000)
PT (s, Median)	1.1(0.8-19)
TB (μmol/L, Median)	1.2(0.8-1.9)
Histological grade	
g1	N=18
g2	N=96
g3	N=85
g4	N=11
Vascular invasion (yes/no)	72/138
Tumor weight (g, Median)	125(20-2924)
Tumor necrosis (%, Median)	5(0-40)
Neoadjuvant treatment (yes/no)	2/208

HCC: hepatocellular carcinoma; BMI: body mass index; AFP: alpha-fetoprotein; PT: prothrombin time; TB: total bilirubin.

**Table 3:** Prognostic factors associated with overall survival in 210 HCC patients.

**Variables**	**Univariate analysis**			**Multivariate analysis**		
	**HR**	**95% CI**	**P value**	**HR**	**95% CI**	**P value**
Gender	0.811	0.438-1.501	0.505	0.863	0.439-1.695	0.669
Age(years)	1.025	0.999-1.052	0.057	1.034	1.004-1.065	0.026*
BMI	1.037	1.007-1.068	0.015*	1.037	1.004-1.070	0.026*
Albumin (g/L)	0.999	0.969-1.029	0.934	0.892	0.634-1.255	0.512
AFP (ng/ml)	1.000	1.000-1.000	0.546	1.000	1.000-1.000	0.122
Platelet (10e9/L)	1.000	1.000-1.000	0.306	1.000	1.000-1.000	0.203
PT (s)	1.044	0.979-1.113	0.188	1.003	0.920-1.094	0.943
TB (μmol/L)	0.040	0.004-0.438	0.008*	0.038	0.002-0.767	0.033*
Histological grade	1.460	0.961-2.218	0.076	1.819	1.132-2.921	0.013*
Vascular invasion	1.610	0.871-2.977	0.129	1.881	0.965-3.667	0.064
Tumor weight	1.000	0.999-1.001	0.658	1.000	0.999-1.001	0.375
Tumor necrosis (%)	1.032	0.984-1.082	0.193	1.043	0.984-1.106	0.159
Neoadjuvant treatment	0.649	0-19426.425	0.646	0	0	0.980
KPNA4	1.001	1.000-1.001	0.027*	1.001	1.000-1.002	0.012*

HCC: hepatocellular carcinoma; CI: confidence interval; HR: hazard ratio; BMI: body mass index; AFP: alpha-fetoprotein; PT: prothrombin time; TB: total bilirubin. *P < 0.05 was considered significant.

### Genetic alterations of KPNA4 and the biological interaction networks in HCC

**Figure 7:**
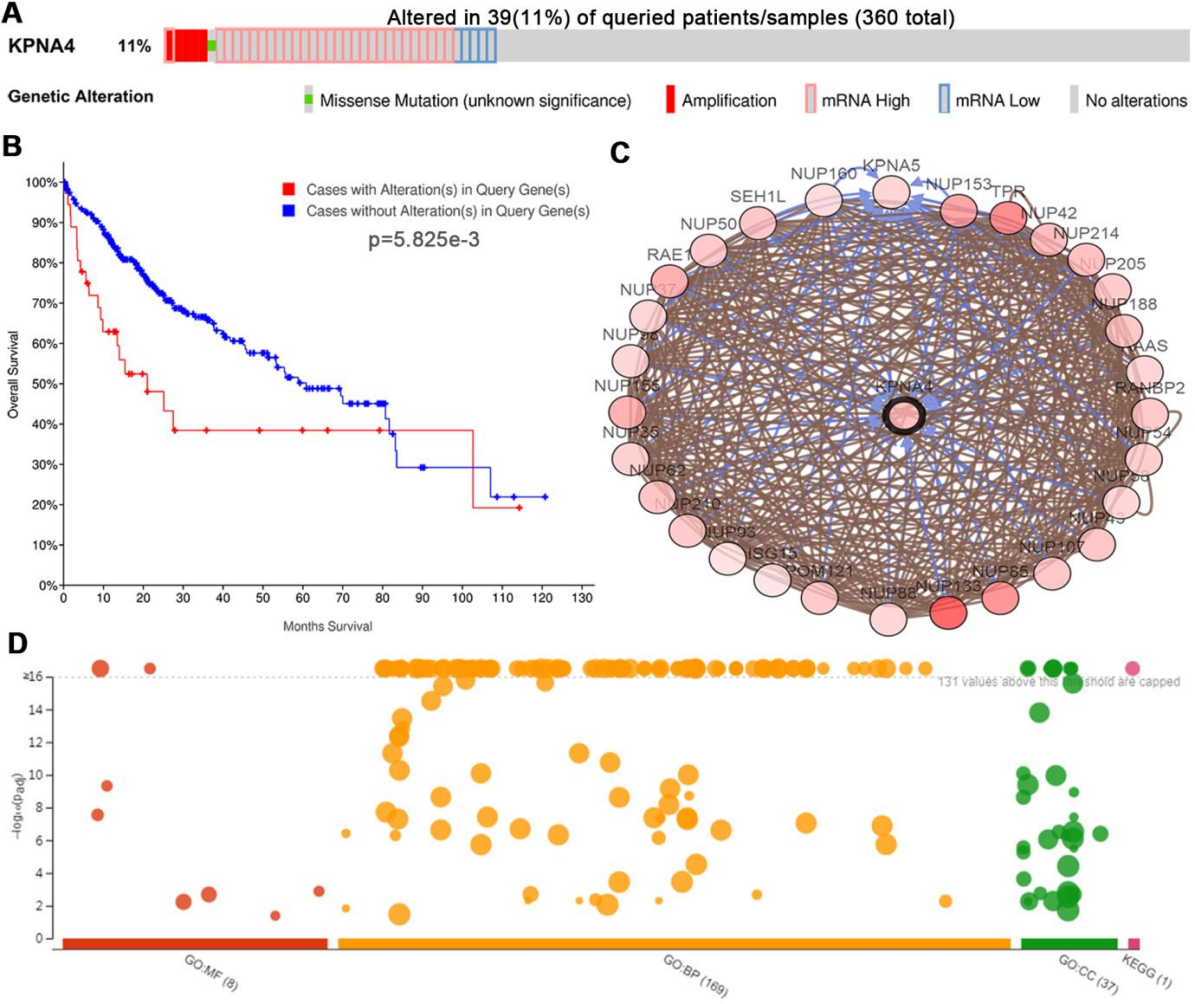
**Genetic mutations of KPNA4 and its associations with OS in HCC patients (cBioPortal).** (**A**) Oncoprint of KPNA4 alterations in HCC. The overview of genomic alterations showed that the mutation rate of KPNA4 was 11%. (**B**) Alterations in KPNA4 poorly affect the prognosis of HCC patients. (**C**) Network view of KPNA4 and its altered neighboring genes in HCC. (**D**) GO functional enrichment and KEGG pathway analyses of KPNA4 and its frequently altered neighboring genes.

To examine KPNA4 alterations and the associations with OS in HCC, data from the cBioPortal database was used. And the results indicated that KPNA4 was altered in 39 of 360 (11%) patients; among them, 27 had high KPNA4 mRNA expression (7.74%), 5 had low KPNA4 mRNA expression (1.43%), 4 had KPNA4 amplification (1.15%), 1 had a KPNA4 mutation (0.29%), and 1 patient had multiple alterations (0.29%) (Figure 7A).

Kaplan-Meier analysis indicated that the KPNA4 alteration was significantly associated with shorter OS (Figure 7B, P = 5.825e-3). Next, neighboring genes that were significantly associated with KPNA4 alterations were used to analyze the biological interaction in cBioPortal, and Figure 7C showed the network. From the results, it could be found that the top 3 mutant genes were NUP133 (36.4%), TPR (23.1%), and NUP85 (21.1%), respectively (Supplementary Table 1). Then, we used the g:Profiler tool to explore functions of KPNA4 and its frequently altered neighboring genes. As shown in Figure 7D and Supplementary Table 2, cellular components, including the host cell, nuclear pore, nuclear envelope, nuclear membrane and nuclear pore outer ring were significantly associated with KPNA4 alterations. Besides, these alterations were noted to be mainly correlated with tRNA export from the nucleus, non-coding RNA export from the nucleus, transport of virus, multiorganism transport and regulation of the glycolytic process. KPNA4 mutation particularly influenced molecular functions, including the structural constituent of the nuclear pore, structural molecule activity, nuclear localization sequence binding, signal sequence binding and nucleocytoplasmic carrier activity. Lastly, KEGG analysis was performed, and it can be found that KPNA4 mutation in HCC was significantly associated with the RNA transport pathway.

### Enrichment analyses of co-expressed genes and kinase, miRNA, and transcription factor targets networks in HCC

**Figure 8:**
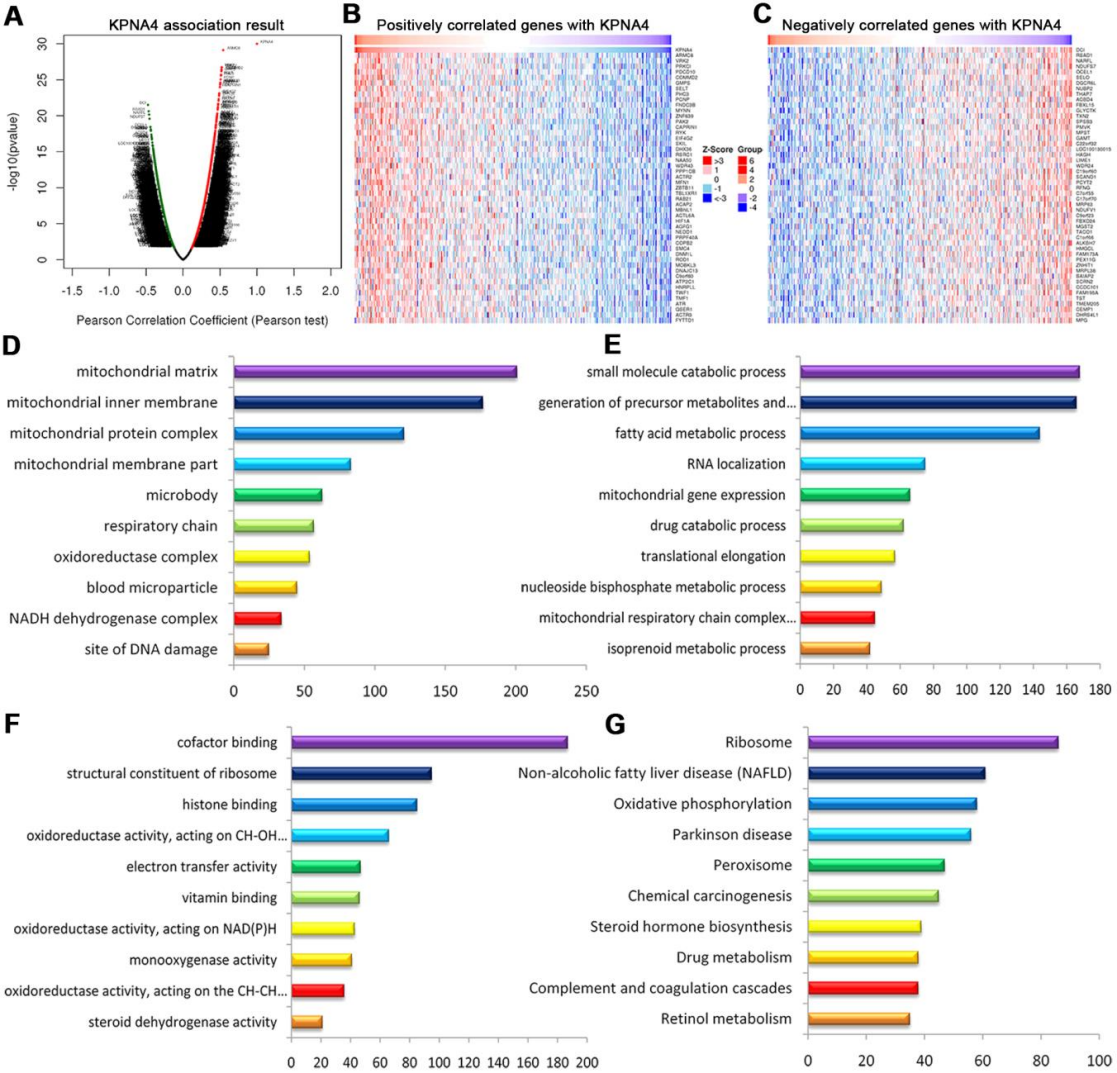
**Genes differentially expressed in correlation with KPNA4 in HCC (LinkedOmics).** (**A**) Volcano plot showing genes correlated with KPNA4 through Pearson’s test analysis. (**B**, **C**) Heat maps showing the top 50 genes positively and negatively correlated with KPNA4 in HCC; red (positive), green (negative). The significantly enriched GO annotations ((**D**) cellular components, (**E**) biological processes, (**F**) molecular functions) and KEGG pathways (**G**) of KPNA4 coexpressed genes were analyzed using GSEA.

To determine the co-expressed genes correlated with KPNA4 in HCC, we employed the LinkedOmics database to perform this analysis. The volcano plot (Figure 8A) illustrated genes which were positively or negatively correlated with KPNA4 (false discovery rate < 0.01). The 50 significant genes which were positively or negatively correlated with KPNA4 were presented by a heat map (Figure 8B, 8C and Supplementary Table 3). Next, the gene set enrichment analysis (GSEA) was used to perform GO term and KEGG analyses, and it could be found that the differentially expressed genes were located primarily in the mitochondria, microbody, respiratory chain, oxidoreductase complex and blood microparticles. These genes were mainly involved in small molecule catabolic processes, the generation of precursor metabolites and energy, fatty acid metabolic processes, RNA localization, and mitochondrial gene expression processes. Moreover, they were involved in cofactor binding, the structural constituents of ribosomes, histone binding, oxidoreductase activity and electron transfer activity (Figure 8D–8F). Finally, enrichment analysis of KEGG showed these genes were primarily involved in ribosome, nonalcoholic fatty liver disease, oxidative phosphorylation, and peroxisome and chemical carcinogenesis pathways (Figure 8G).

Furthermore, we constructed the kinase, miRNA and transcription factor target networks by GSEA to explore the targets of KPNA4 in HCC. As indicated in Supplementary Figure 2A, it could be seen that cyclin-dependent kinase 2 (CDK2), CDK1, mitogen-activated protein kinase 1 (MAPK1), ataxia-telangiectasia mutated (ATM), and checkpoint kinase 1 (CHEK1) were the top 5 significant kinase target networks. (GCACTTT) MIR-17-5P, MIR-20A, MIR-106A, MIR-106B, MIR-20B, MIR-519D, (TTTGCAC) MIR-19A, MIR-19B and (TGTTTAC) MIR-30A-5P, MIR-30C, MIR-30D, MIR-30B, MIR-30E-5P were the most significant miRNA target networks (Supplementary Figure 2B). Besides, heat shock transcription factor-like HSF1_01, transcription factor SOX9_B1, and the E2F transcription factor (E2F) family, including E2F_Q4_01, E2F_Q4, and E2F_02 were the main transcription factor target networks (Supplementary Figure 2C).

### Expression of KPNA4 is correlated with HCC prognosis

**Figure 9:**
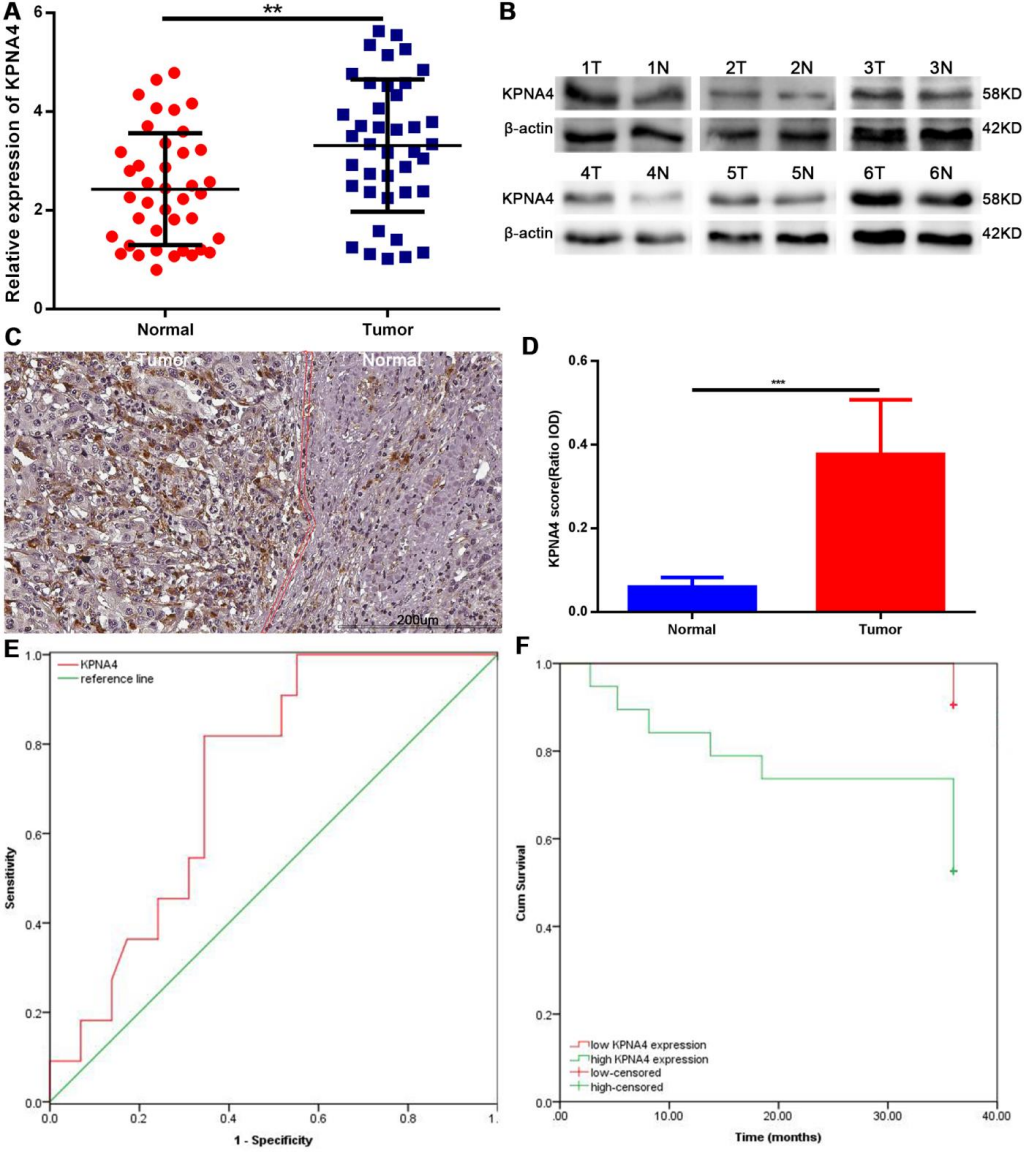
**Expression levels of KPNA4 and prognostic values in HCC patients.** (**A**) KPNA4 mRNA level was increased in HCC tissues compared with normal tissues as measured by qRT-PCR. (**B**) KPNA4 protein level was increased in HCC tissues compared with normal tissues as measured by western blot. (**C**) KPNA4 expression by immunohistochemistry staining. (**D**) Semi-quantitative analysis of KPNA4 protein expression between cancerous specimens and non-cancerous parts. (**E**) ROC of KPNA4 level in HCC showing that elevated KPNA4 level correlated with HCC incidence. (**F**) High KPNA4 expression level correlated with poor OS.

To confirm the conclusions arrived at above, we conducted qRT-PCR assay to measure KPNA4 mRNA level in 40 pairs of HCC tissues and corresponding normal tissues. The clinical features of included cases were concluded in Supplementary Table 4. Our results indicated KPNA4 level was higher in HCC tissue than that in normal tissue (Figure 9A, P < 0.01). Next, western blotting and immunohistochemistry (IHC) assays were used to evaluate KPNA4 protein levels in these samples, and it could be found that KPNA4 protein level was higher in HCC than that in normal tissue (Figure 9B–9D).

Finally, we performed the receiver operating characteristic (ROC) curve analysis and Kaplan-Meier analysis to determine potential application value of KPNA4 in HCC; and found that KPNA4 may serve as a biomarker to diagnose HCC (area under the ROC curve [AUC] = 0.726, 95% CI: 0.569-0.882, P = 0.029; Figure 9E), and to predict patient prognosis (Figure 9F, P = 0.005).

## DISCUSSION

HCC is a relatively common disease, and numerous methods currently have been used in the treatment of HCC. However, because there are no reliable methods of early detection and because the postoperative recurrence rate of HCC is high, outcomes remain poor.

A number of different components are transported via karyopherin mediation [9–11]. Karyopherin-α (or importin-α) plays an essential role in this process by combining molecules which contain specific nuclear localization signals (NLSs) [12–14]. KPNA4 plays critical roles in the nuclear localization of many biological processes [15–17], and it has been identified as a therapeutic target in a number of different malignancies [6–8, 18]. However, few studies have examined KPNA4 with respect to HCC.

Herein, we first examined the correlations of KPNA4 level and immune infiltration cells, and then detected KPNA4 expression in common human tumor tissues and normal tissues. Although KPNA4 mRNA expressions were at relatively low levels in both HCC tissue and normal liver tissue, the protein level was higher in the liver compared with that in other organs. This may result from that KPNA4 protein translation efficiency in the liver is much greater than that in other organs; surely, what can be confirmed is that additional studies are needed to clearly identify mechanisms that regulate KPNA4. We next studied differences of KPNA4 expression in HCC tissue and compared normal liver tissue, and results from multiple databases all indicated that KPNA4 mRNA and protein expressions in normal liver tissue are significantly lower than that in HCC, a result consistent with that of previous studies [6, 8].

Infiltrating lymphocytes have been shown to be prognostic factors for numerous cancers [19, 20]. In present study, we identified that KPNA4 level was correlated with certain infiltrating cells and immune markers; however, further study is needed to determine how immune cells affect KPNA4 expression and promote the growth and development of HCC.

Further, we performed Kaplan-Meier analysis using a group of HCC patients identified in TCGA database, and found that KPNA4 expression was negatively correlated with patient prognosis. Additionally, genetic alterations of KPNA4 had a significant effect on patient survival. Complete data from 210 patients identified in TCGA database was used to perform Cox analyses, and the results showed that KPNA4 expression, as well as age, BMI, TB level, and tumor histopathological grade were significant prognostic factors for HCC. These results further demonstrated the value of KPNA4 for HCC patients. Finally, HCC and normal tissues from patients treated at our center were studied and the results were consistent with those of the database analyses.

Recent studies have shown that inhibition of KPNA4 attenuates prostate cancer metastasis [6] and that KPNA4 participates in the proliferation of cutaneous squamous cell carcinoma [8]. In current study, increased KPNA4 expression was significantly associated with higher tumor stage and grade.

Genetic alterations and dysregulated amplification are believed to be vital for the development of many tumors [21–23]*.* Epigenetic changes are viewed as very important in many malignancies [24, 25], as well as in HCC [1, 26, 27]. Our results identified genetic mutations in KPNA4 in HCC, and the mutation was significantly associated with patient survival. In addition, the functional networks showed that the neighboring genes, together with KPNA4, generally participated in the transport of cargoes, including tRNA and viruses via the RNA transport pathway, a finding that is consistent with the inherent characteristic of the KPNA family as nuclear transporters.

Results from present study showed that the functional network of KPNA4 is mainly involved in the mitochondria, microbody, respiratory chain and cell cycle. Functional network of KPNA4 transcription was associated with genomic stability, gene expression and the cell cycle. These results indicate that KPNA4 has roles in cell cycle regulation and cell function regulation, which provides additional evidence that alterations of the KPNA4 gene may be associated with the development of malignancies, including HCC.

There are limitations of this study that must be acknowledged. Comparisons between KPNA4 and other common biomarkers of HCC, such as alpha fetoprotein (AFP), des-γ-carboxyprothrombin (DCP) and glypican-3 were not performed because data were not abundantly available in public databases. Second, as a novel potential biomarker, more research work is still needed for KPNA4's testing methods and clinical application in HCC. Third, experimental evidences of KPNA4 promoting the growth and progression of HCC are still indispensable.

## CONCLUSIONS

Collectively, this study indicated that KPNA4 actively participate in the pathogenesis of HCC, and that it could be viewed as a potential diagnostic and prognostic marker.

## MATERIALS AND METHODS

### Bioinformatics analysis

The Tumor IMmune Estimation Resource (TIMER, https://cistrome.shinyapps.io/timer/) is used to explore the correlation between gene expression and immune infiltration in various cancers [28]. Here, KPNA4 expression was analyzed in HCC patients, and B lymphocytes, CD8^+^ T lymphocytes, CD4^+^ T lymphocytes, macrophages, neutrophils, and dendritic cells were included for the analysis. Multivariable Cox analysis was performed to investigate the clinical relevance of tumor immune subsets with common clinical factors and KPNA4 expression in HCC. The Gene Expression Profiling Interactive Analysis (GEPIA, http://gepia.cancer-pku.cn/) was used to examine the associations of KPNA4 level and markers of T cell exhaustion [29].

KPNA4 expression in human tissues and protein subcellular localization were validated using the Human Protein Atlas (HPA) (https://www.proteinatlas.org) database [30]. In addition, KPNA4 expression was further investigated in the Metabolic gEne RApid Visualizer (MERAV, http://merav.wi.mit.edu) database [31] and the HCCDB database (http://lifeome.net/database/hccdb) [32]. Meanwhile, co-expression networks in HCC tissue, adjacent liver tissue, and normal liver tissue were constructed using the HCCDB.

KPNA4 mRNA expression and its associations with various clinical indicators were explored using the UALCAN database (http://ualcan.path.uab.edu) [33]. Kaplan-Meier plotter (http://kmplot.com/analysis/) analysis was conducted to investigate prognostic value of KPNA4 for HCC patients.

Genetic alterations of KPNA4 in HCC and its correlation with patient survival were analyzed using the cBio Cancer Genomics Portal database (http://cbioportal.org) [34]. In addition, neighboring genes of KPNA4 were then used to perform Gene Ontology (GO) and Kyoto Encyclopedia of Genes and Genomes (KEGG) pathway analyses using the g:Profiler database (http://biit.cs.ut.ee/gprofiler/) [35]. The correlated genes, together with KPNA4, were analyzed in HCC using data from a LinkedOmics cohort (n=371) (http://www.linkedomics.org/login.php) [36]. GO, KEGG, kinase-target enrichment, miRNA-target enrichment, and transcription factor-target enrichment analyses of the identified correlated genes were further performed using the Web-based GEne SeT AnaLysis Toolkit (WebGestalt) [37].

### Patients and tissue samples

In present study, 40 pairs of HCC tissues and corresponding normal tissues were obtained from HCC patients who underwent hepatectomy at the Third Affiliated Hospital of Sun Yat-Sen University, Guangzhou, China. The protocol of this study was approved by the Ethics Committee of the Third Affiliated Hospital of Sun Yat-Sen University, and each patient has signed the informed consent.

### Quantitative reverse transcription- polymerase chain reaction (qRT-PCR)

Total RNA of all clinical samples in our center was extracted using the TRIzol reagent (Invitrogen, Carlsbad, CA, USA). Then, we conducted reverse transcription assays using the GoScript™ System (Promega, Madison, WI, USA). Finally, quantitative polymerase chain reaction (q-PCR) analysis was performed using SYBR-green PCR Master Mix and a Roche PCR 480 system (Roche Applied Science, Germany). The following primer sequences for this assay were used: KPNA4 (forward): 5′-CAGGAGATTCTTCCAGCCCTTTGTGT-3′ and (reverse): 5′-ATTACCATCTGTATTTGTTCATTGCCAGCATC-3′; glyceraldehyde 3-phosphate dehydrogenase (GAPDH) (forward): 5′-GGAGCGAGATCCCTCCAAAAT-3′ and (reverse): 5′- GGCTGTTGTCATACTTCTCATGG-3′.

### Western blotting

Total protein was extracted using an EpiQuik Nuclear Extraction Kit (Epigentek, Farmingdale, NY, USA). A BCA Protein Assay Kit (Pierce, Rockford, IL, USA) was used to quantify the protein concentration. Equal protein quantities were separated by 10% SDS-PAGE gel and then transferred onto nitrocellulose membranes (0.2 μm). Incubation of antibodies against KPNA4 (Santa Cruz, CA, USA) and β-actin (Cell Signaling Technology, Danvers, MA, USA) was performed overnight at 4° C. Then, incubation of HRP-conjugated secondary antibody (Abcam, Cambridge, MA, USA) was performed. Finally, an enhanced chemiluminescence kit (ECL; Pierce) was used to visualize target protein expression.

### Immunohistochemistry (IHC) analysis

IHC assays were performed using formalin-fixed HCC tissue samples which was embedded in 4-μm-thick paraffin sections. The sections were incubated with antibody against KPNA4 (Santa Cruz,CA, USA) overnight at 4° C, and then the secondary antibody (Abcam) for 1 h at room temperature. ImagePro Plus 6.0 software was used to perform semiquantitative scoring of the images.

### Cox regression analysis

Cox proportional hazards regression models were adopted to determine the association of KPNA4 expression with patient survival. The SPSS version 22.0 software was used in this study, and differences were considered significant with values of P < 0.05.

## Supplementary Material

Supplementary Figures

Supplementary Table 1

Supplementary Table 2

Supplementary Table 3

Supplementary Table 4

## References

[1] Forner A, Reig M, Bruix J. Hepatocellular carcinoma. Lancet. 2018; 391:1301–14. 10.1016/S0140-6736(18)30010-2 29307467

[2] Ikenberg K, Valtcheva N, Brandt S, Zhong Q, Wong CE, Noske A, Rechsteiner M, Rueschoff JH, Caduff R, Dellas A, Obermann E, Fink D, Fuchs T, et al. KPNA2 is overexpressed in human and mouse endometrial cancers and promotes cellular proliferation. J Pathol. 2014; 234:239–52. 10.1002/path.4390 24930886

[3] Mortezavi A, Hermanns T, Seifert HH, Baumgartner MK, Provenzano M, Sulser T, Burger M, Montani M, Ikenberg K, Hofstädter F, Hartmann A, Jaggi R, Moch H, et al. KPNA2 expression is an independent adverse predictor of biochemical recurrence after radical prostatectomy. Clin Cancer Res. 2011; 17:1111–21. 10.1158/1078-0432.CCR-10-0081 21220479

[4] Rachidi SM, Qin T, Sun S, Zheng WJ, Li Z. Molecular profiling of multiple human cancers defines an inflammatory cancer-associated molecular pattern and uncovers KPNA2 as a uniform poor prognostic cancer marker. PLoS One. 2013; 8:e57911. 10.1371/journal.pone.0057911 23536776 PMC3607594

[5] Sakai M, Sohda M, Miyazaki T, Suzuki S, Sano A, Tanaka N, Inose T, Nakajima M, Kato H, Kuwano H. Significance of karyopherin-{alpha} 2 (KPNA2) expression in esophageal squamous cell carcinoma. Anticancer Res. 2010; 30:851–56. 20393006

[6] Yang J, Lu C, Wei J, Guo Y, Liu W, Luo L, Fisch G, Li X. Inhibition of KPNA4 attenuates prostate cancer metastasis. Oncogene. 2017; 36:2868–78. 10.1038/onc.2016.440 27941876 PMC5436935

[7] Wang H, Tao T, Yan W, Feng Y, Wang Y, Cai J, You Y, Jiang T, Jiang C. Upregulation of miR-181s reverses mesenchymal transition by targeting KPNA4 in glioblastoma. Sci Rep. 2015; 5:13072. 10.1038/srep13072 26283154 PMC4539550

[8] Zhang M, Luo H, Hui L. MiR-3619-5p hampers proliferation and cisplatin resistance in cutaneous squamous-cell carcinoma via KPNA4. Biochem Biophys Res Commun. 2019; 513:419–25. 10.1016/j.bbrc.2019.03.203 30967266

[9] Görlich D, Kutay U. Transport between the cell nucleus and the cytoplasm. Annu Rev Cell Dev Biol. 1999; 15:607–60. 10.1146/annurev.cellbio.15.1.607 10611974

[10] Mattaj IW, Englmeier L. Nucleocytoplasmic transport: the soluble phase. Annu Rev Biochem. 1998; 67:265–306. 10.1146/annurev.biochem.67.1.265 9759490

[11] Ström AC, Weis K. Importin-beta-like nuclear transport receptors. Genome Biol. 2001; 2:REVIEWS3008. 10.1186/gb-2001-2-6-reviews3008 11423015 PMC138946

[12] Lange A, Mills RE, Lange CJ, Stewart M, Devine SE, Corbett AH. Classical nuclear localization signals: definition, function, and interaction with importin alpha. J Biol Chem. 2007; 282:5101–05. 10.1074/jbc.R600026200 17170104 PMC4502416

[13] Hood JK, Silver PA. Cse1p is required for export of Srp1p/importin-alpha from the nucleus in Saccharomyces cerevisiae. J Biol Chem. 1998; 273:35142–46. 10.1074/jbc.273.52.35142 9857050

[14] Kutay U, Bischoff FR, Kostka S, Kraft R, Görlich D. Export of importin alpha from the nucleus is mediated by a specific nuclear transport factor. Cell. 1997; 90:1061–71. 10.1016/s0092-8674(00)80372-4 9323134

[15] Agrawal T, Gupta GK, Agrawal DK. Calcitriol decreases expression of importin α3 and attenuates RelA translocation in human bronchial smooth muscle cells. J Clin Immunol. 2012; 32:1093–103. 10.1007/s10875-012-9696-x 22526597 PMC3444658

[16] Fagerlund R, Kinnunen L, Köhler M, Julkunen I, Melén K. NF-{kappa}B is transported into the nucleus by importin {alpha}3 and importin {alpha}4. J Biol Chem. 2005; 280:15942–51. 10.1074/jbc.M500814200 15677444

[17] Sachan N, Mishra AK, Mutsuddi M, Mukherjee A. The drosophila importin-α3 is required for nuclear import of notch in vivo and it displays synergistic effects with notch receptor on cell proliferation. PLoS One. 2013; 8:e68247. 10.1371/journal.pone.0068247 23840889 PMC3698139

[18] Ahluwalia A, Jones MK, Tarnawski AS. Key role of endothelial importin-α in VEGF expression and gastric angiogenesis: novel insight into aging gastropathy. Am J Physiol Gastrointest Liver Physiol. 2014; 306:G338–45. 10.1152/ajpgi.00382.2013 24356884

[19] Azimi F, Scolyer RA, Rumcheva P, Moncrieff M, Murali R, McCarthy SW, Saw RP, Thompson JF. Tumor-infiltrating lymphocyte grade is an independent predictor of sentinel lymph node status and survival in patients with cutaneous melanoma. J Clin Oncol. 2012; 30:2678–83. 10.1200/JCO.2011.37.8539 22711850

[20] Ohtani H. Focus on TILs: prognostic significance of tumor infiltrating lymphocytes in human colorectal cancer. Cancer Immun. 2007; 7:4. 17311363 PMC2935759

[21] Kim J, Reber HA, Dry SM, Elashoff D, Chen SL, Umetani N, Kitago M, Hines OJ, Kazanjian KK, Hiramatsu S, Bilchik AJ, Yong S, Shoup M, Hoon DS. Unfavourable prognosis associated with K-ras gene mutation in pancreatic cancer surgical margins. Gut. 2006; 55:1598–605. 10.1136/gut.2005.083063 16682430 PMC1860104

[22] Renehan AG. Cumulative incidence of metachronous colorectal cancer risk for mismatch repair gene mutation carriers is overestimated. Gut. 2012; 61:783. 10.1136/gutjnl-2011-300997 21873468

[23] Wei F, Lin CC, Joon A, Feng Z, Troche G, Lira ME, Chia D, Mao M, Ho CL, Su WC, Wong DT. Noninvasive saliva-based EGFR gene mutation detection in patients with lung cancer. Am J Respir Crit Care Med. 2014; 190:1117–26. 10.1164/rccm.201406-1003OC 25317990 PMC5447327

[24] Huang X, Yan J, Zhang M, Wang Y, Chen Y, Fu X, Wei R, Zheng XL, Liu Z, Zhang X, Yang H, Hao B, Shen YY, et al. Targeting epigenetic crosstalk as a therapeutic strategy for EZH2-aberrant solid tumors. Cell. 2018; 175:186–99.e19. 10.1016/j.cell.2018.08.058 30220457

[25] Mohammad HP, Barbash O, Creasy CL. Targeting epigenetic modifications in cancer therapy: erasing the roadmap to cancer. Nat Med. 2019; 25:403–18. 10.1038/s41591-019-0376-8 30842676

[26] El Tayebi HM, Abdelaziz AI. Epigenetic regulation of insulin-like growth factor axis in hepatocellular carcinoma. World J Gastroenterol. 2016; 22:2668–77. 10.3748/wjg.v22.i9.2668 26973407 PMC4777991

[27] Li C, Li R, Zhang W. Progress in non-invasive detection of liver fibrosis. Cancer Biol Med. 2018; 15:124–36. 10.20892/j.issn.2095-3941.2018.0018 29951337 PMC5994553

[28] Li T, Fan J, Wang B, Traugh N, Chen Q, Liu JS, Li B, Liu XS. TIMER: a web server for comprehensive analysis of tumor-infiltrating immune cells. Cancer Res. 2017; 77:e108–10. 10.1158/0008-5472.CAN-17-0307 29092952 PMC6042652

[29] Tang Z, Li C, Kang B, Gao G, Li C, Zhang Z. GEPIA: a web server for cancer and normal gene expression profiling and interactive analyses. Nucleic Acids Res. 2017; 45:W98–102. 10.1093/nar/gkx247 28407145 PMC5570223

[30] Asplund A, Edqvist PH, Schwenk JM, Pontén F. Antibodies for profiling the human proteome-the human protein atlas as a resource for cancer research. Proteomics. 2012; 12:2067–77. 10.1002/pmic.201100504 22623277

[31] Shaul YD, Yuan B, Thiru P, Nutter-Upham A, McCallum S, Lanzkron C, Bell GW, Sabatini DM. MERAV: a tool for comparing gene expression across human tissues and cell types. Nucleic Acids Res. 2016; 44:D560–66. 10.1093/nar/gkv1337 26626150 PMC4702927

[32] Lian Q, Wang S, Zhang G, Wang D, Luo G, Tang J, Chen L, Gu J. HCCDB: a database of hepatocellular carcinoma expression atlas. Genomics Proteomics Bioinformatics. 2018; 16:269–75. 10.1016/j.gpb.2018.07.003 30266410 PMC6205074

[33] Sighoko D, Curado MP, Bourgeois D, Mendy M, Hainaut P, Bah E. Increase in female liver cancer in the Gambia, West Africa: evidence from 19 years of population-based cancer registration (1988-2006). PLoS One. 2011; 6:e18415. 10.1371/journal.pone.0018415 21490972 PMC3072390

[34] Guy J, Peters MG. Liver disease in women: the influence of gender on epidemiology, natural history, and patient outcomes. Gastroenterol Hepatol (N Y). 2013; 9:633–39. 24764777 PMC3992057

[35] Cauble S, Abbas A, Balart L, Bazzano L, Medvedev S, Shores N. United States women receive more curative treatment for hepatocellular carcinoma than men. Dig Dis Sci. 2013; 58:2817–25. 10.1007/s10620-013-2731-9 23812858 PMC8717408

[36] Singal AG, Chan V, Getachew Y, Guerrero R, Reisch JS, Cuthbert JA. Predictors of liver transplant eligibility for patients with hepatocellular carcinoma in a safety net hospital. Dig Dis Sci. 2012; 57:580–86. 10.1007/s10620-011-1904-7 21953138

[37] Li T, Fan J, Qin LX, Zhou J, Sun HC, Qiu SJ, Ye QH, Wang L, Tang ZY. Risk factors, prognosis, and management of early and late intrahepatic recurrence after resection of primary clear cell carcinoma of the liver. Ann Surg Oncol. 2011; 18:1955–63. 10.1245/s10434-010-1540-z 21240562

