# Supplementary material for: Value of KPNA4 as a diagnostic and prognostic biomarker for hepatocellular carcinoma
Source: Aging (Albany NY). 2021 Feb 1;13(4):5263–83. doi: 10.18632/aging.202447 (PMC7950262; doi:10.18632/aging.202447)
Supplement: Supplementary Figures [file aging-13-202447-s001.pdf]

SUPPLEMENTARY FIGURES

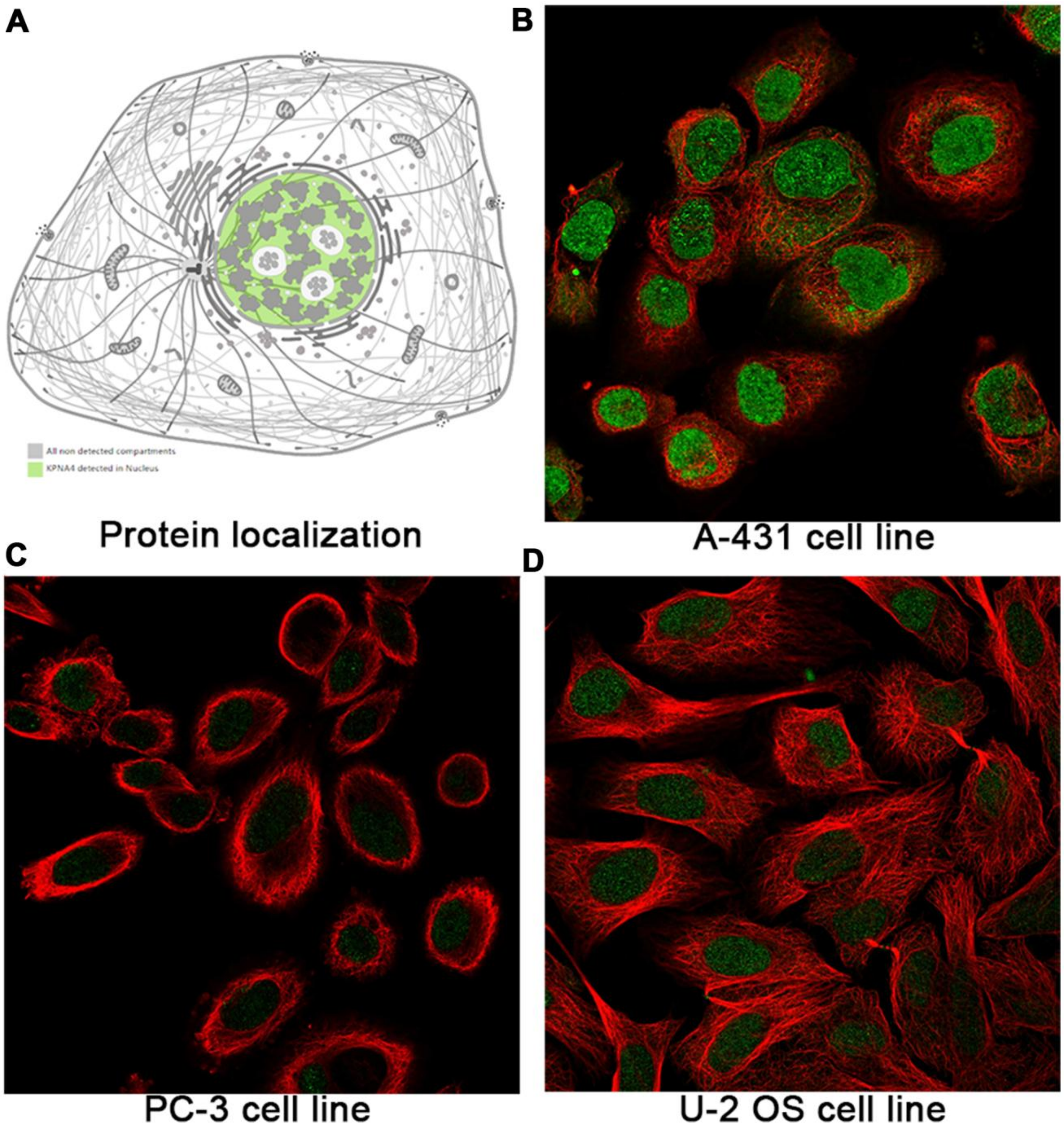

Supplementary Figure 1. Subcellular localization of KPNA4 in HPA database. (**A**–**D**) KPNA4 protein subcellular localization in A-431, PC-3 and U-2 OS cell lines.

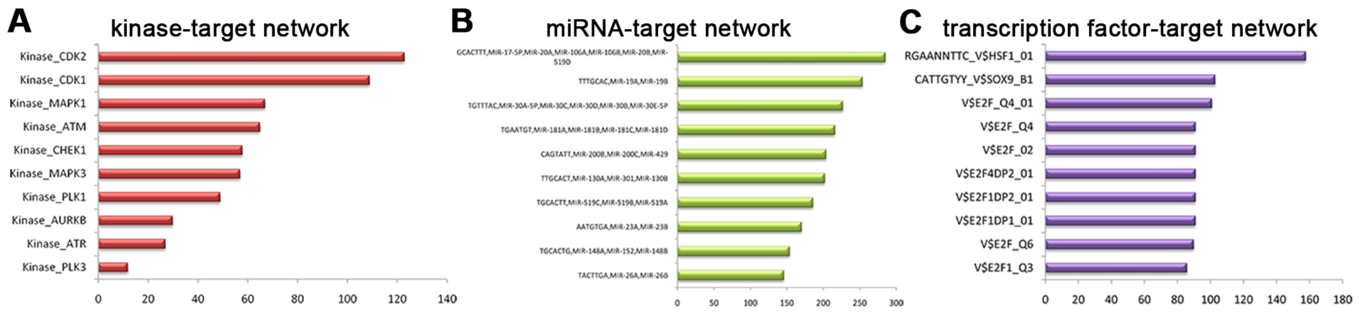

Supplementary Figure 2. Genes differentially expressed in correlation with KPNA4 in HCC (LinkedOmics). Significantly enriched KPNA4 networks of kinase (**A**), miRNA (**B**) or transcription factor (**C**) targets of KPNA4 coexpressed genes in HCC were analyzed using GSEA.
