# Supplementary material for: Value of KPNA4 as a diagnostic and prognostic biomarker for hepatocellular carcinoma
Source: Aging (Albany NY). 2021 Feb 1;13(4):5263–83. doi: 10.18632/aging.202447 (PMC7950262; doi:10.18632/aging.202447)
Supplement: Supplementary Table 1 [file aging-13-202447-s002.pdf]

Supplementary Table 1. Neighboring genes associated with KPNA4 mutation.

Genes	Amplification	Homozygous deletion	Up-regulation	Down-regulation	Mutation	Total alteration
NUP133	0.089		0.303	0.008	0.025	0.364
TPR	0.089		0.164	0.003	0.014	0.231
NUP85	0.056		0.181	0	0.006	0.211
NUP153	0.042		0.133	0	0.017	0.164
RAE1	0.014		0.128	0.006	0.003	0.142
NUP155	0.014		0.119	0	0.011	0.136
NUP42	0.014		0.1	0.014	0	0.117
KPNA4	0.014		0.081	0.014	0.003	0.108
NUP214	0.006		0.058	0.031	0.025	0.108
NUP62	0.008		0.086	0	0.006	0.1
NUP43	0.003	0.019	0.069	0.006	0.006	0.097
SEH1L	0.011		0.064	0.028	0	0.097
NUP107	0.019		0.081	0.003	0	0.094
NUP188	0.003		0.081	0	0.014	0.094
NUP50	0.006	0.003	0.083	0.003	0.003	0.092
POM121	0.011		0.078	0	0.003	0.092
RANBP2	0.003	0.003	0.047	0.011	0.028	0.092
NUP210	0.008		0.075	0	0.011	0.089
NUP205	0.011		0.075	0	0.006	0.086
NUP54	0.003	0.003	0.036	0.044	0	0.081
NUP35	0.008	0.006	0.067	0.006	0	0.078
AAAS	0.003		0.058	0.006	0.003	0.069
KPNA5		0.014	0.056	0	0	0.069
NUP37	0.006		0.061	0	0.006	0.069
NUP88		0.019	0.031	0.019	0.008	0.069
NUP58	0.003	0.003	0.047	0.011	0.003	0.067
NUP98	0.006	0.003	0.033	0.022	0.008	0.067
NUP160	0.003		0.053	0	0.006	0.058
NUP93	0.003	0.006	0.031	0	0.008	0.047
ISG15	0.003	0.031	0.008	0	0	0.042
