# Supplementary material for: Value of KPNA4 as a diagnostic and prognostic biomarker for hepatocellular carcinoma
Source: Aging (Albany NY). 2021 Feb 1;13(4):5263–83. doi: 10.18632/aging.202447 (PMC7950262; doi:10.18632/aging.202447)
Supplement: Supplementary Table 4 [file aging-13-202447-s005.pdf]

Supplementary Table 4. Clinicopathological characteristics of 40 HCC patients.

Characteristics	HCC patients (N=40)
Gender (male/female)	37/3
Age (years, mean±SD)	49.18±12.44
ALT (U/L, mean±SD)	54±61.98
ALB (g/L, mean±SD)	42.83±3.5
PT (s, mean±SD)	13.51±1.163
HBsAg (positive/negative)	40/0
AFP (ng/ml, median)	144 (1.7-5451)
ECOG PS score (0/1)	33/7
Child-Pugh grade (A/B)	30/10
BCLC stage (0, A/B, C)	23/17
Tumor size (cm, mean±SD)	5.286±3.437
Capsule integrity (yes/no)	31/9
Satellite nodule (yes/no)	2/38
Liver cirrhosis (yes/no)	27/13
MVI (yes/no)	3/37
Anatomical hepatectomy (yes/no)	20/20
Blood loss (ml, < 500/≥ 500)	36/4
3-year survival (yes/no)	11/29

HCC: hepatocellular carcinoma; ALT: alanine aminotransferase; ALB: albumin; PT: prothrombin time; HBsAg: hepatitis B surface antigen; AFP: alpha-fetoprotein; ECOG: Eastern Cooperative Oncology Group; PS: performance status; BCLC: Barcelona clinic liver cancer; MVI: microvascular invasion.
